# Long-Lasting Growth Hormone Regulated by the Ubiquitin-Proteasome System

**DOI:** 10.3390/ijms22126268

**Published:** 2021-06-10

**Authors:** Myung-Sun Kim, Kyunggon Kim, Su Kyung Oh, Gidae Lee, Jin-Ock Kim, Lan Li, Jung-Hyun Park, Kwang-Hyun Baek

**Affiliations:** 1 UbiProtein Corp., Seongnam-si 13493, Korea; lg-kms@hanmail.net (M.-S.K.); pomnagea@hanmail.net (S.K.O.); gdlee1129@gmail.com (G.L.); 2 Asan Institute for Life Sciences, Asan Medical Center, Seoul 05505, Korea; kimkyunggon@gmail.com; 3 Department of Convergence Medicine, University of Ulsan College of Medicine, Seoul 05505, Korea; 4 Clinical Proteomics Core Laboratory, Convergence Medicine Research Center, Asan Medical Center, Seoul 05505, Korea; 5 Bio-Medical Institute of Technology, Asan Medical Center, Seoul 05505, Korea; 6 Department of Biomedical Science, CHA University, Seongnam-si 13488, Korea; kjo8909@gmail.com (J.-O.K.); dreamerlanlan@gmail.com (L.L.); jhpark6237@nate.com (J.-H.P.); 7 School of Basic Medical Sciences, North China University of Sciences and Technology, Tangshan 063210, China

**Keywords:** anti-ubiquitination technology, half-life, hGH, ubiquitination

## Abstract

To increase the half-life of growth hormones, we proposed its long-lasting regulation through the ubiquitin-proteasome system (UPS). We identified lysine residues (K67, K141, and K166) that are involved in the ubiquitination of human growth hormone (hGH) using ubiquitination site prediction programs to validate the ubiquitination sites, and then substituted these lysine residues with arginine residues. We identified the most effective substituent (K141R) to prevent ubiquitination and named it AUT-hGH. hGH was expressed and purified in the form of hGH-His, and ubiquitination was first verified at sites containing K141 in the blood stream. Through the study, we propose that AUT-hGH with an increased half-life could be used as a long-lasting hGH in the blood stream.

## 1. Introduction

The human growth hormone (hGH) is synthesized as 22 kDa polypeptide and stored in the anterior pituitary gland by somatotroph cells [1]. It acts either directly or indirectly on various tissues and physiological systems such as longitudinal bone, skeletal muscle, liver, total body nitrogen balance, and so on [2]. A recombinant hGH (rhGH) is produced as a 191-amino acid protein that acts as an endocrine hormone, stimulating growth, cell reproduction, and regeneration [3]. This rhGH has been used clinically since 1985 for the treatment of children’s growth disorders caused by a growth hormone deficiency, chronic renal failure, Turner’s syndrome, and adult growth hormone deficiency [4]. Some of the leading global human growth hormones available commercially are Biotropin (Lifetech Labs, Hong Kong), Genotropin (Pfizer Inc., New York, NY, USA), Norditropin (Norvo Nordisk Inc., Plainsboro, NJ, USA), Humatrope (Eli Lilly and Company, Indianapolis, IN, USA), Saizen (Merck Serono, Darmstadt, Germany), Hypertropin (Neogenica bioscience Ltd., China), Ansomone (AnkeBio Co., Anhui, China), Serostim (EMD Serono Inc., Rockland, MA, USA), Jintropin (GeneScience Pharmaceuticals Co., Jilin, China) and Hygetropin (Hygene BioPharm Co., China). However, the rhGH therapy should be performed daily or three times a week, because these drugs have a short half-life in the blood circulation due to low uptake by proteolytic system enzymes. The therapy causes several problems including weak compatibility, renal toxicity, and an increase in treatment cost for the patient [4]. Therefore, a lot of studies have been conducted in recent years with the aim of increasing the half-life of the drugs. For a sustained release of rhGH, a lot of formulations have been used. Examples of approaches include coating of a monomolecular layer of positively charged poly arginine with hGH for crystalline formulation [5], protein encapsulation into polymeric microspheres [6,7], injectable hydrogels [8,9,10], and degradable implants [11,12]. Moreover, various protein fusion techniques including PEGylation (where PEG stands for polyethylene glycol), binding with albumin or antigen-binding fragment (Fab), and fusion with other proteins, have recently been attempted to develop sustained growth hormones [13]. Representative products using these techniques are GX-H9 [14,15], MOD-4023 [16,17] LAPSrhGH [13] AG-B1512 [13], Somapacitan [18,19,20], Jintrolong [21,22], and so on. However, these methods cannot be used for the production of some protein pharmaceuticals due to the large size of the fusion protein, which may result in decreased productivity and instability. In this study, the mechanism of protein degradation that occurs when hGH circulates in the body is evaluated. 

In eukaryotic cells, proteins and peptides degrade in two ways, lysosomal or through ubiquitin-proteasome system (UPS) [23]. About 80% of proteins degrade through UPS, which regulates the degradation of most cellular proteins in eukaryotes and presides over protein turnover and homeostasis in vivo [23]. The ubiquitin, a key protein of UPS, is a small protein consisting of 76 highly conserved amino acids in all eukaryotic cells [23]. For the selective degradation of a protein in eukaryotic cells, E1 (ubiquitin-activating), E2 (ubiquitin-conjugating) and E3 (ubiquitin-ligase) enzymes act in continuation to promote the ubiquitination of the target proteins. The ubiquitin-tagged proteins are then decomposed by the 26S proteasome of ATP-dependent protein degradation complex [23,24]. Recently, it has been reported that extracellular ubiquitin is found at nanomolar concentrations in human plasma and serum [25]. Plasma ubiquitin levels are elevated during hairy cell leukemia (HCL), allergy, autoimmune infections, and other disorders [26]. Moreover, extracellular ubiquitin plays the role of an immune modulator affecting T and B lymphocytes [27] and is involved in the regulation of the immune system [25]. Extracellular ubiquitination regulates the initiation, propagation, and termination of immune responses such as intracellular ubiquitination [25].

In this study, we confirmed the ubiquitination of hGH in blood for the first time and constructed an effective substituent (K141R) to prevent the ubiquitination of hGH via an in vitro assay. This suggests that AUT-hGH with an increased half-life might be a long-lasting hGH in the blood stream.

## 2. Results

### 2.1. hGH Mutants Are Less Ubiquitinated

**Figure 1:**
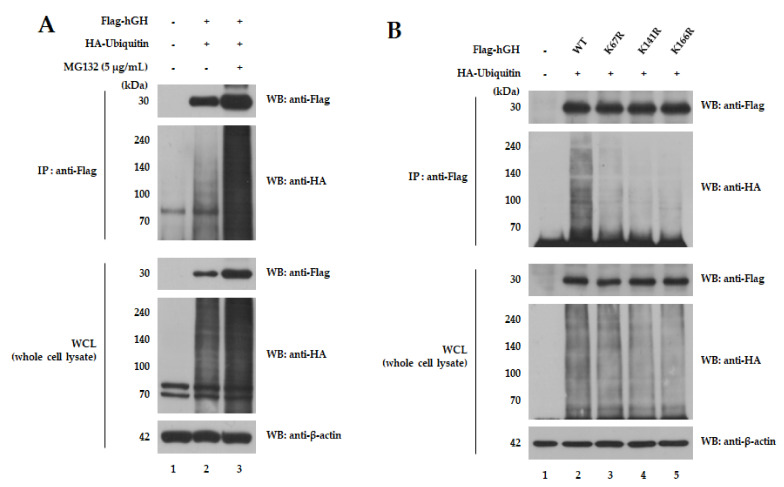
The ubiquitination of hGH and its mutants in cells. (**A**) Ubiquitination of hGH. 293T cells were transfected with Flag-hGH and HA-ubiquitin together. Ubiquitination of hGH was confirmed by co-immunoprecipitation with an anti-Flag antibody and the counter blot was detected by an anti-HA antibody. hGH forms a poly ubiquitin chain and thus increases it after treatment with MG132 (proteasome inhibitor, 5 μg/mL) for 6 h. (**B**) Ubiquitination of hGH mutants. The 293T cells were transfected with each Flag-hGH (K67R), hGH (K141R) and hGH (K166R) and HA-ubiquitin together. Ubiquitination level was checked by co-immunoprecipitation with an anti-Flag antibody and immunoblotting with an anti-HA antibody. Mutants of hGH create less poly ubiquitin chain formation compared to the WT.

For these experiments, we used the hGH precursor form. The numbering of mutations was also based on the precursor form (Supplementary Figure S1). To investigate whether hGH is ubiquitinated in the cells, we performed a ubiquitination assay (Figure 1A). In overexpressed cells of hGH with the Flag-tag, poly ubiquitin chain formation was observed (Figure 1A, lane 2), which increased after treatment with MG132 (proteasome inhibitor, 5 μg/mL) for 6 h (Figure 1A, lane 3). These smear bands indicate poly ubiquitin chain formation, and more intense bands indicate increased poly ubiquitin chain formation (Figure 1A, lane 3). Moreover, to find a site that can effectively prevent the ubiquitination of hGH, its mutant forms were constructed (Supplementary Table S1), and a ubiquitination assay was performed (Figure 1B). It can be observed that the smear bands that indicate poly ubiquitin chain formation are less intense in hGH (K67R), hGH (K141R) and hGH (K166R) mutants as compared to the wild-type (WT) (Figure 1B, lanes 3–5). These results suggest that a smaller amount of ubiquitin was detected because the ubiquitin did not bind to the mutant hGH. These results indicate that ubiquitin first binds to an hGH, and then a poly ubiquitin chain is formed, and finally the hGH is degraded by the UPS.

### 2.2. hGH (K141R) Extends Its Half-Life

**Figure 2:**
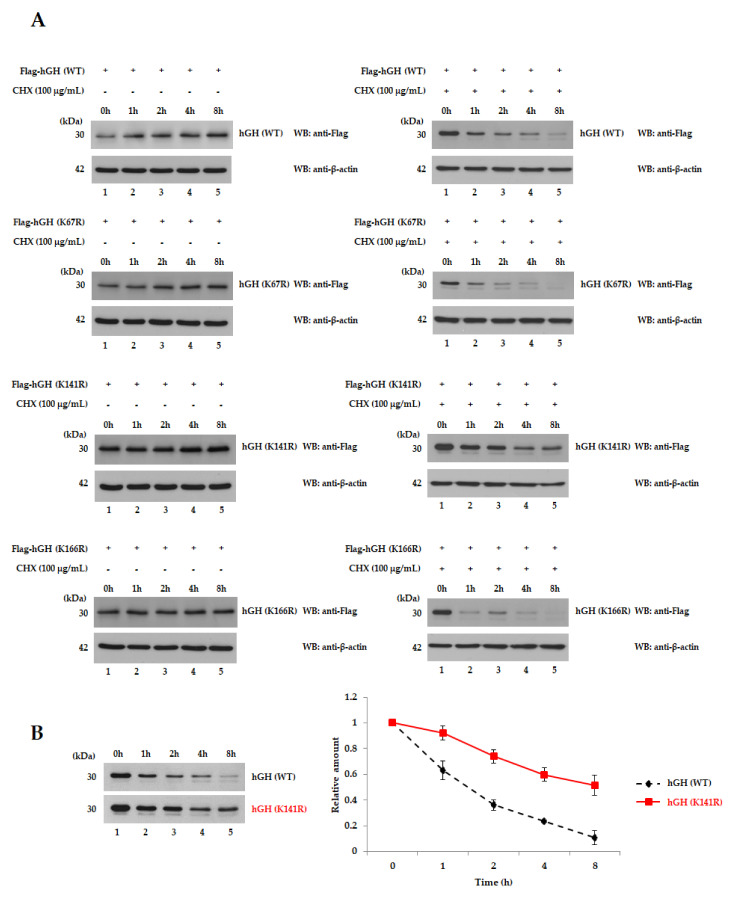

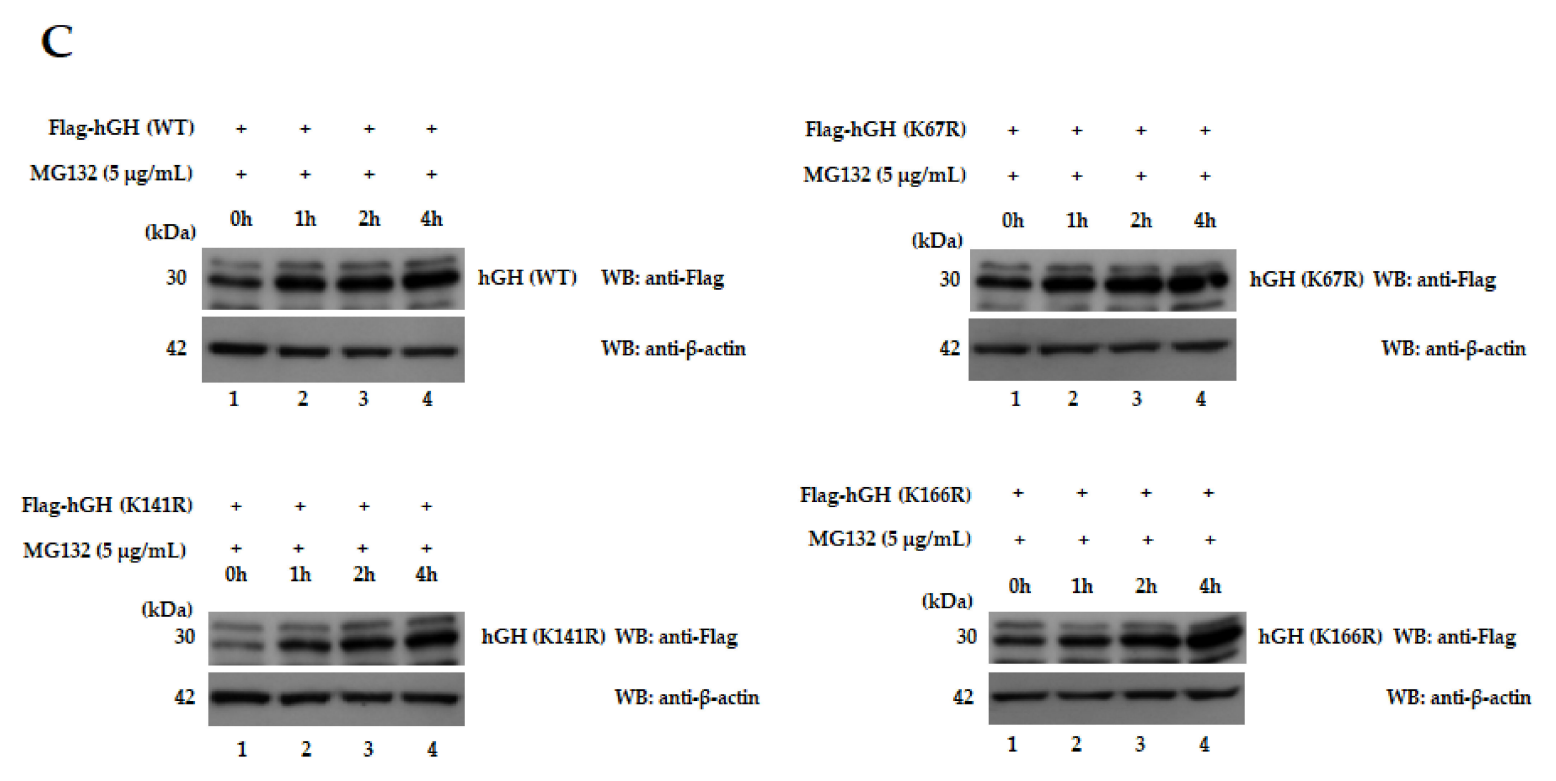
Half-life test of hGH and its mutants in cells. (**A**) hGH (K141R) increased the half-life. 293T cells were individually transfected with Flag-hGH (K67R), hGH (K141R) and hGH (K166R). At 24 h following transfection, CHX was treated at a concentration of 100 μg/mL for 0, 1, 2, 4, and 8 h, and then cell lysates were prepared. The presence of hGH protein was detected using an anti-Flag antibody. The half-life of hGH (K141R) was prolonged to 8 h or more, while the half-life of hGH (WT) was less than 2 h. (**B**) The intensity of the band between hGH (WT) and hGH (K141R) was relatively quantified and is shown as a graph. (**C**) Proteasome inhibition of hGH increased in a time-dependent manner with the treatment of MG132.

To verify whether hGH mutants extend their half-life, the hGH mutants K67R, K141R, and K166R were used (Supplementary Table S1). To confirm the half-life of every protein, we checked the bands that indicate hGH at 0, 1, 2, 4 and 8 h after the treatment with protein synthesis inhibitor and cycloheximide (CHX) (100 μg/mL) (Figure 2A). Hence, the half-life of hGH (K141R) was prolonged to 8 h or more, while the half-life of hGH (WT) was less than 2 h (Figure 2A). The intensity of the band was relatively quantified and shown as a graph (Figure 2B). In contrast, the hGH expression levels increased in a time-dependent manner, because proteasome inhibition occurred with the treatment of MG132 (Figure 2C). These results show that not all lysines that block ubiquitination increase the half-life. Therefore, this indicates that the K141 site of hGH plays a key role in extending its half-life. Subsequently, hGH (K141R) that increased the half-life of hGH was named AUT-hGH.

### 2.3. AUT-hGH and hGH Were Purified in Their Untagged Form

**Figure 3:**
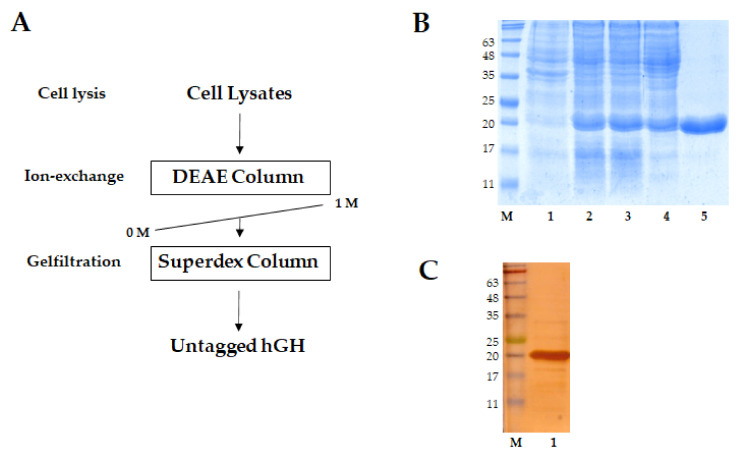
Purification of recombinant AUT-hGH. (**A**) Scheme of untagged AUT-hGH purification from *E. coli*. The purification process was followed by cell lysis, ion-exchange, and gel filtration. (**B**) Purification of untagged AUT-hGH. At each step, elutions from each column were separated by 15% sodium dodecyl sulfate-polyacrylamide (SDS-PAGE) gel and analyzed by Coomassie blue staining. M indicates molecular weight marker; lane 1 indicates total protein before isopropyl β-D-1-thiogalactopyranoside (IPTG) induction; lane 2 shows the soluble fraction after IPTG induction and cell lysis; lane 3 shows soluble fraction before passing the diethylaminoethyl (DEAE) column for ion-exchange; lane 4 shows soluble fraction after passing DEAE column for ion-exchange; lane 5 shows purified untagged AUT-hGH after passing Superdex column for gel filtration. hGH was also purified by the same process. (**C**) Silver staining of 6 μg of purified untagged AUT-hGH. M, molecular weight marker; lane 1, untagged AUT-hGH.

Protein expression at low temperatures often significantly improves the solubility of recombinant proteins [28,29,30]. Therefore, we tried several conditions for the culture of hGH and production of hGH and AUT-hGH. In this study, recombinant AUT-hGH and hGH were induced at 20 °C and the amount of soluble hGH and AUT-hGH increased without tagging (Figure 3). The results show that their molecular weights were approximately 22.26 kDa (hGH (WT); expected: 22.246 kDa) and 22.28 kDa (AUT-hGH; expected: 22.246 kDa). We observed a major band, indicating high purity obtained by silver staining (Figure 3C).

### 2.4. Structure and Biological Activity of hGH and AUT-hGH

**Figure 4:**
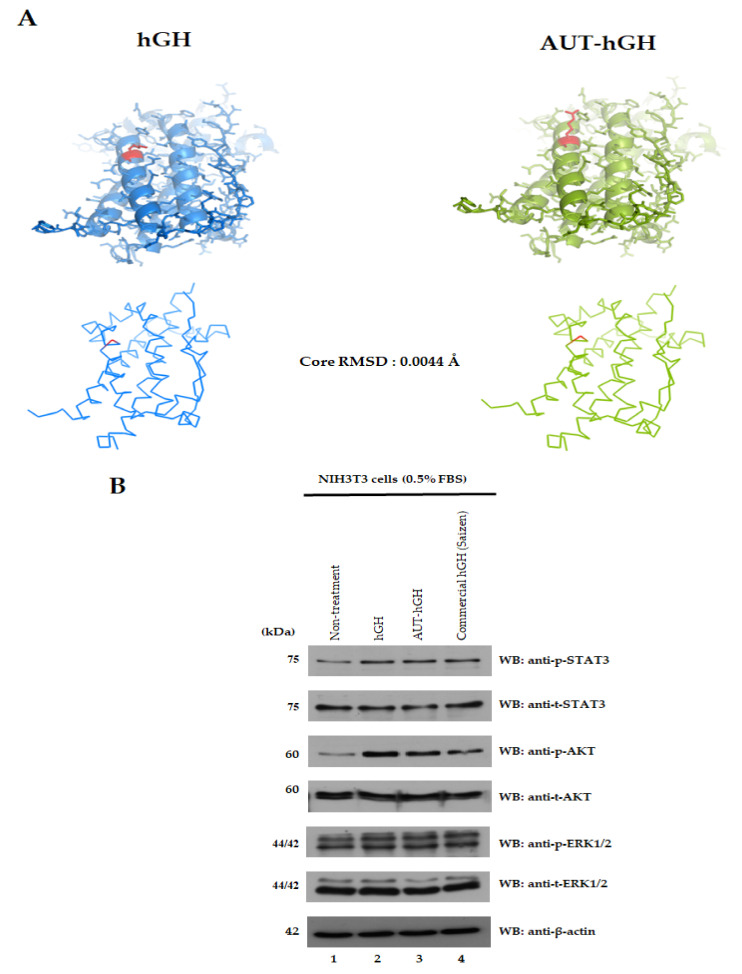
Comparison of structure and biological activity between hGH and AUT-hGH. (**A**) Structural similarity between hGH and AUT-hGH. The core RMSD score of 0.0044Å shows that AUT-hGH has a structure similar to hGH. Red positions indicate K141 and R141 in hGH and AUT-hGH, respectively. (**B**) Signal transduction of purified AUT-hGH in NIH3T3 cells. Starved NIH3T3 cells were treated with 100 ng/mL concentrations of hGH, AUT-hGH, and commercial hGH for 24 h. After 2 days of treatment, phospho-STAT3, phospho-AKT, and phospho-ERK 1/2 proteins were detected using an anti-p-STAT3, anti-p-AKT, and anti-p-ERK1/2 antibodies. The counter blots were detected by antibodies against proper total forms and β-actin. AUT-hGH induced phospho-STAT3 and phospho-AKT in NIH3T3 cells like hGH and commercial hGH.

A structural comparison of hGH and AUT-hGH was performed to detect whether there is any difference between the WT hGH and AUT-hGH (Figure 4A). Root mean square deviation (RMSD) is commonly used to measure the similarity between two superimposed atomic coordinates [31]. Lysine 141 is in a stable alpha-helix motif and substitution to arginine was simulated using in silico method. There is no structural hindrance and no change in Ramachandran Plot after substitution using WinCoot (ver. 0.9.4.1) [32] and Calphacarbon RMSD between the two structures was 0.0044Å, indicating that the substitution of lysine to arginine in 141 residue does not vary the secondary structure.

To determine whether the purified hGH and AUT-hGH were biologically active, we verified the signal transduction in the NIH3T3 cells. It has been reported that the growth hormone controls the transcription of STAT protein [33], and mediators in GH signaling pathway include STATs and PI3K-AKT [34]. In this study, we treated rhGH and AUT-hGH, and used commercial hGH (Saizen) as a positive control. NIH3T3 cells were starved for 24 h and then treated with hGH, AUT-hGH, and commercial hGH at a concentration of 100 ng/mL. After 2 days of treatment, their signal transduction was evaluated by Western blot analysis (Figure 4B). AUT-hGH showed intense phospho-STAT3, and phospho-AKT in NIH3T3 cells similar to hGH and commercial hGH. Hence, AUT-hGH maintains biological activity in NIH3T3 cells.

### 2.5. hGH Ubiquitination in the Blood Stream

**Figure 5:**
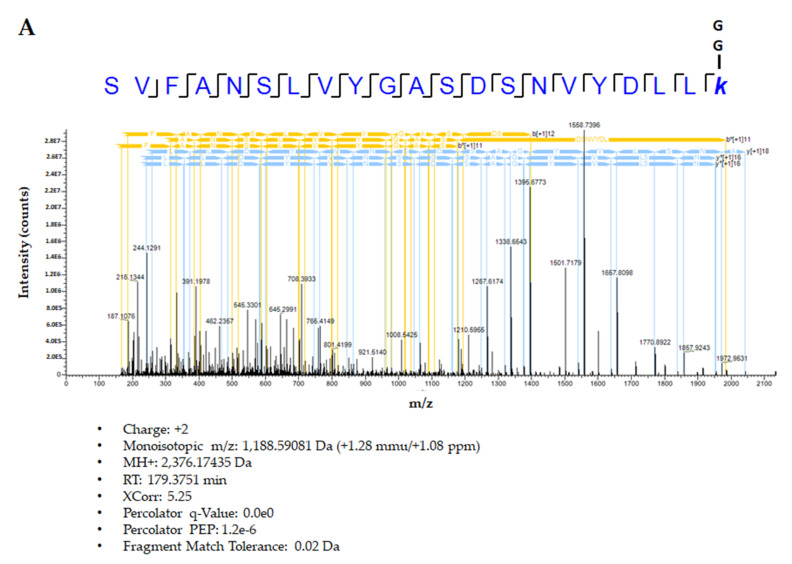

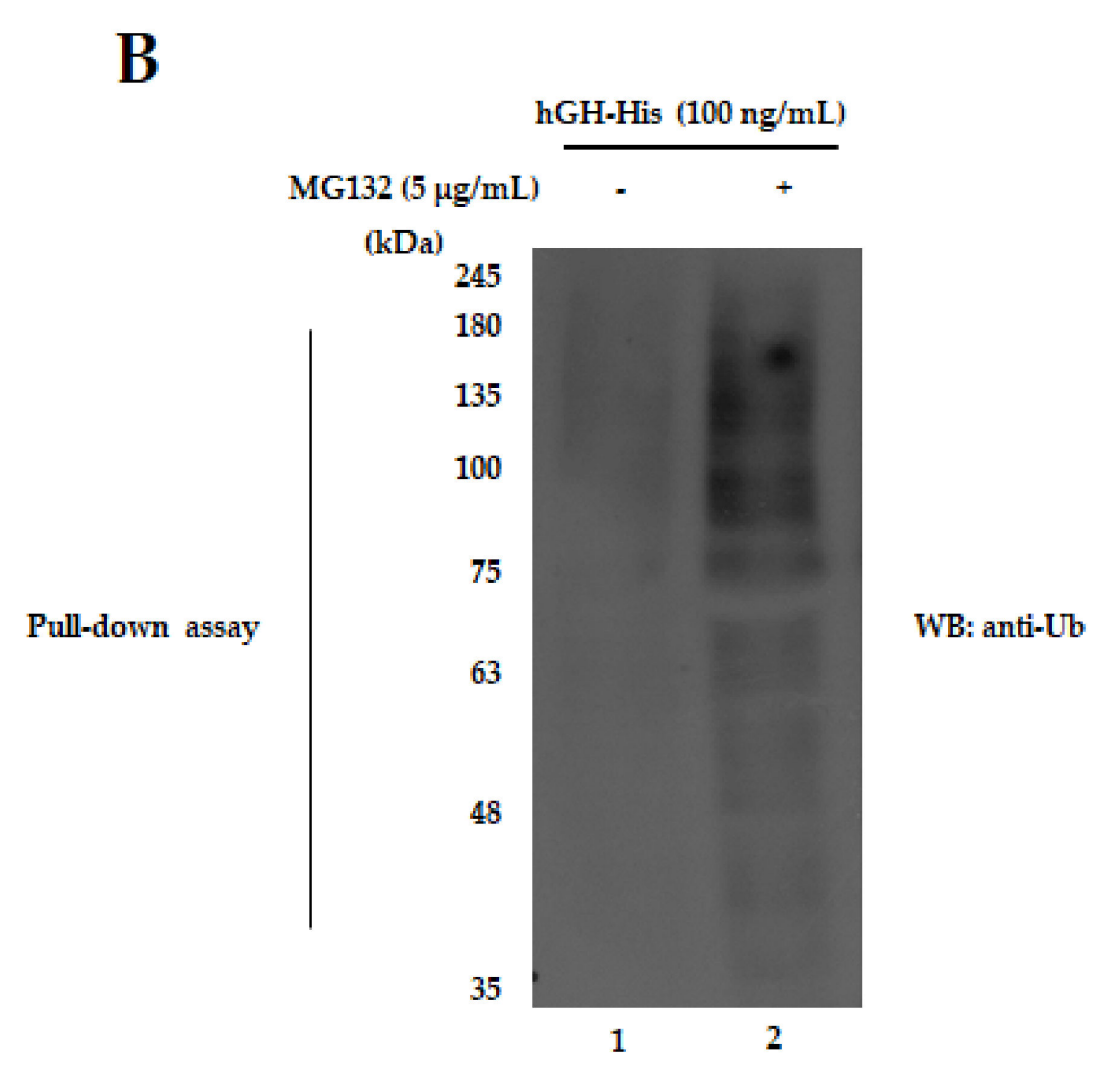
(**A**) MS analysis of ubiquitination for hGH in the blood stream. The hGH at K141 residue is modified to be Gly Gly at epsilon-amine, which is a significant modification of ubiquitination. (**B**) Polyubiquitination of hGH in the blood stream. hGH-His was incubated for 1 h at 37 °C in blood. Lane 1 indicates 100 ng/mL hGH-His without MG132; lane 2 shows 100 ng/mL hGH-His with MG132.

We checked whether hGH is ubiquitinated in blood, because it has been reported that that extracellular ubiquitin is found at nanomolar concentrations in human plasma and serum [25,26,27]. To confirm the ubiquitination of hGH in blood, purified hGH-His was incubated for 1 h in a mouse whole blood sample. Subsequently, a pull-down assay was performed on hGH-His using nickel nitrilotriacetic acid (Ni-NTA) beads, and mass spectrometry (MS) and Western blot analysis were performed to identify ubiquitination (Figure 5 and Supplementary Table S2). Hypothetically, precipitated hGH-His from mouse blood is exposed to the ubiquitination system of mouse blood and a lysine residue is obtained, which is modified to Gly Gly at epsilon-amine. This is a significant modification of ubiquitination. The spectrum of hGH indicates the clear isopeptide of glycine with a K141 residue (Figure 5A). Furthermore, Western blot analysis using an anti-ubiquitin antibody showed that hGH undergoes polyubiquitination in the blood stream, and MG132 increases the band smear (Figure 5B). This is the first report that hGH is polyubiquitinated in the blood stream. It was confirmed again that if the lysine at 141 of hGH was substituted with arginine, the binding of ubiquitin with hGH would be prevented.

### 2.6. Increase in AUT-hGH Half-Life in the Blood Stream

**Figure 6:**
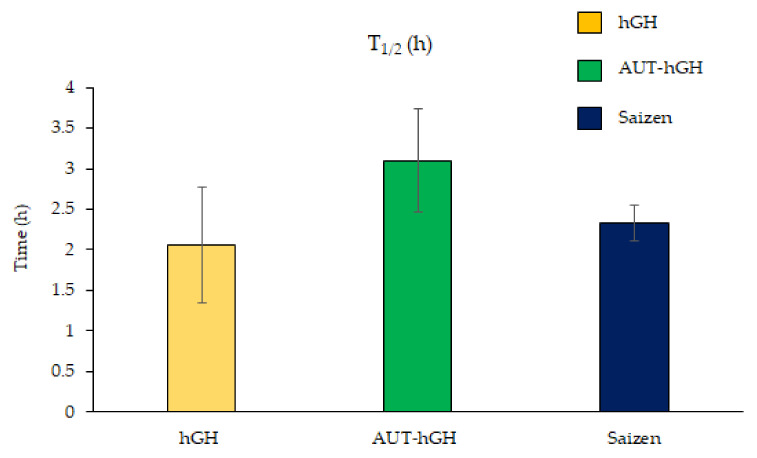
The pharmacokinetic graphs of the hGH, AUT-hGH, and the Saizen. The half-life of the hGH, AUT-hGH, and the Saizen was analyzed. The half-life of AUT-hGH seems to be the longest.

The half-life of AUT-hGH increases in the blood stream. In the study, hGH, AUT-hGH, and commercial hGH (Saizen) were subcutaneously administrated to male Sprague Dawley (SD) rats. The blood was collected at 0, 1, 2, 4, 8, 12, 16, 24, 30, and 48 h after administration, and the plasma was separated. Using the plasma, we measured the hGH protein in the blood stream using an enzyme-linked immunosorbent assay (ELISA) (Figure 6). The graph shows the pharmacokinetic graphs of the hGH, AUT-hGH, and the Saizen (Figure 6). The plasma half-life of the AUT-hGH increases approximately 1.5-fold compared to hGH, and 1.33-fold compared to the Saizen. 

### 2.7. Maintaining the Efficacy of AUT-hGH 

**Figure 7:**
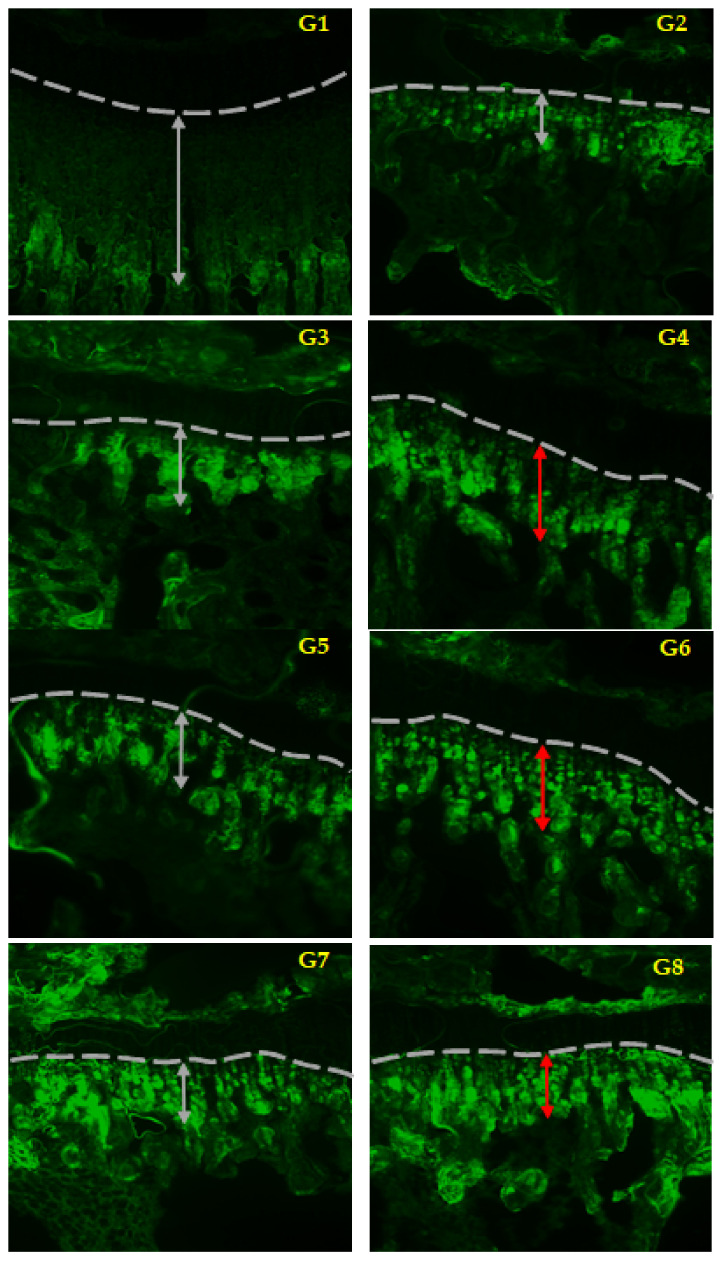
Maintaining the efficacy of AUT-hGH. The white and red arrows indicate the distance between the growth plate and the band formed by tetracycline. G1 indicates a normal rat, and G2 is a rat with its pituitary gland removed. G3 and G4 indicate rats which were daily administered, G5 and G6 are rats which were administered at intervals of 3 days, and G7 and G8 are those administered at intervals of 7 days. G3, G5, and G7 were administrated hGH, and G4, G6, and G8 were administrated AUT-hGH.

To check the efficacy of AUT-hGH, rats with their pituitary gland removed at 4 weeks of age were administered hGH and AUT-hGH on a daily basis, and in intervals of 3 days and 7 days, repeatedly. Normal mice were used as a positive control, and mice that were not treated with hGH were used as a negative control. To measure the tibia length, we checked that the distance between growth plate and the band formed by tetracycline (Figure 7). As a result, the negative control group was significantly decreased compared to the normal control group (Figure 7, G1 and G2). In SD rats which were daily administrated with hGH and AUT-hGH, the bone growth rate significantly increased compared to the negative control group (Figure 7, G3 and G4). In contrast, in SD rats which were administrated with hGH and AUT-hGH at intervals of 3 days and 7 days, hGH and AUT-hGH maintained a similar growth promoting effect (Figure 7, G5–G8).

## 3. Discussion

Because growth hormone is received by subcutaneous injection every day, patients require convenience. Therefore, diverse methods for the development of long-lasting hGH have been suggested. As a slow-release product, Genentech first launched Neutrofind Depot., and LG Life Science released Diclase in 2007 [13]. However, the sustained-release therapeutic agent causes an unwanted immune response in the human body, causes pain due to the use of a thick syringe, and is not economically viable due to a low production yield. Neutropin Depot was withdrawn from the market because it was less effective than the first generation. Additionally, fused sugar chain engineering with polyethylene glycol as a polymer is used to increase endurance. However, highly concentrated PEG, used as a protein stabilizer, has been reported to be unsafe. SonoVue (a commercial contrast agent containing PEG) may cause anaphylactic shock, and polysorbate 80 (PEG-containing polymer) may cause urticarial shock. Anti-PEG also increases drug abstinence, poses a risk of subsequent infusion reactions, and increases tissue fears in animals treated with PEGylated protein [35,36,37,38,39,40,41]. Fusion with other proteins enables the development of long-lasting hGH, which is used as anti-human serum albumin Fab antibody, immunoglobulin Fc portion of IgD and IgG4, and carboxyl-terminal peptide (CTP) of hCG β-subunit [13]. However, these methods have not been used as a universal half-life increasing method due to various reasons such as lack of economic efficiency at a low production yield, occurrence immune reactions with long-term use, and the toxicity of chemicals used in the binding process. Therefore, these methods are currently being studied to find solutions for these limitations and problems.

To find solutions to address these limitations and problems, we explored a mechanism of protein degradation that naturally occurs when hGH circulates in the body. Here, we confirmed that hGH is ubiquitinated in the blood stream, and K141 of hGH plays a role in the ubiquitination. Therefore, the AUT-hGH that changed the K141 to R141 caused a reduction in ubiquitination and an increase in half-life. To confirm the polyubiquitination of hGH in the blood stream (Figure 5B), we first considered the presence of ATP, E1 (ubiquitin-activating), E2 (ubiquitin-conjugating), and E3 (ubiquitin-ligase) enzymes in the blood stream. ATP is present in human plasma [26]. Recently, according to the human plasma proteome database that summarizes the results of analysis on human blood proteins (http://www.plasmaproteomedatabase.org/, accessed on 10 May 2021), proteasome and ubiquitin-related enzymes of units of ng/mL are present in plasma. Therefore, it is meaningful to state that the polyubiquitination of hGH occurs in blood.

Moreover, because we only substituted one amino acid, there was little change in the tertiary structure of the protein, and no change in protein size. Hence, patients do not experience pain, as in the case of fusion with other proteins. AUT-hGH did not maximize its half-life in the body; this limitation can be overcome by using existing technologies. Future developments in AUT technology will introduce these benefits. For example, we plan to combine AUT technology with the existing long-lasting technology, which has several limitations and problems, and suggest AUT-hGH-PEG, AUT-hGH-CTP, and so on. These trials may enable the development of more effective long-lasting protein-based drugs.

## 4. Materials and Methods

### 4.1. hGH and Its Mutants for Expression

#### 4.1.1. Preparation of hGH in Mammalian Cells

The *hGH* DNA amplified by a polymerase chain reaction (PCR) was treated with Eco RI, and then ligated to pCS4-Flag vector [42], previously digested with the same enzyme. The PCR conditions are as follows: Step 1: at 94 °C for 3 min (1 cycle); Step 2: at 94 °C for 30 s; at 60 °C for 30 s; at 72 °C for 30 s (25 cycles); and Step 3: at 72 °C for 10 min (1 cycle), and then held at 4 °C.

#### 4.1.2. Isolation and Substitution of Lysine (K) Residue

To determine lysine residues involved in the ubiquitination process, we used prediction of ubiquitination sites with Bayesian Discriminant Method (http://bdmpub.biocuckoo.org/prediction. php, accessed on 10 May 2021) with “high sensitivity” as a performance selection.

For hGH mutants, lysine residues were replaced with arginine (R) using site-directed mutagenesis. The following primer sets were used for PCR to produce the mutated plasmid DNAs. For hGH K67R, fwd 5′-CCAAAGGAACAGAGGTATTCATTC-3′, rev 5′-CAG GAATGAATACCTCTGTTCCTT-3′; for hGH K141R, fwd 5′-GACCTCCTAAGGGACCTAGAG-3′, rev 5′-CTCTAGGTCCCTTAGGAGGTC-3′; and for hGH K166R, fwd 5′-CAGATCTTCAGGCAGACCTAC-3′, rev 5′-GTAGGTCTGCCTGAAGATCTG-3′. Three mutant plasmid DNAs from which one lysine residue was replaced by arginine (K->R) were produced using pCS4-Flag-hGH as a template (Supplementary Table S2).

### 4.2. Cell Lines

NIH3T3 (mouse embryo fibroblast cell line, ATCC CRL-1658, Manassas, VA, USA) and HEK 293T cell (human embryonic kidney cell line, ATCC CRL-11268) were grown in Dulbecco’s modified Eagle’s medium (DMEM, GIBCO BRL, Rockville, MD, USA) supplemented with 10% fetal bovine serum (FBS, GIBCO BRL) and 1% penicillin and streptomycin (GIBCO BRL).

### 4.3. Ubiquitination Analysis in Cells

The HEK 293T cells were counted, seeded using SOL COUNT (SOL Inc., Seoul, Korea) into 1 × 10^7^ cells in 100-mm culture plate, and transfected with the plasmid encoding pCS4-Flag-hGH and pMT123-HA-ubiquitin [43]. To check the ubiquitination level of hGH and its mutants, the HEK 293T cells were co-transfected with 1 μg of pMT123-HA-ubiquitin DNA and 4 μg of pCS4-Flag-hGH, pCS4-Flag-hGH (K67R), pCS4-Flag-hGH (K141R) and pCS4-Flag-hGH (K166R). At 24 h after the transfection, the cells were treated with MG132 (Sigma-Aldrich, M7449, Darmstadt, Germany) (proteasome inhibitor, 5 μg/mL) for 6 h, after which immunoprecipitation analysis was carried out.

The sample obtained for the immunoprecipitation was dissolved in a buffer solution (1% Triton X, 150 mM NaCl, 50 mM Tris-HCl (pH 8.0), and 1 mM PMSF) and was then mixed with anti-Flag (Sigma-Aldrich, F3165). Subsequently, the mixture was incubated at 4 °C overnight. The immunoprecipitant was separated following the reaction with A/G bead at 4 °C for 2 h. Then, the separated immunoprecipitant was washed twice with buffering solution. The protein sample was separated by SDS-PAGE, after mixing with 2× SDS buffer and heating at 100 °C for 7 min. The separated protein was transferred to the polyvinylidene difluoride membrane (PVDF, Millipore, Darmstadt, Germany), and an anti-HA antibody (sc-7392, Santa Cruz Biotechnology, Santa Cruz, CA, USA) was used for Western blotting. Blots were detected using ECL reagent solution (Western blot detection kit, Ab Frontier, Seoul, Korea).

### 4.4. Analysis of Half-Life Using Protein Synthesis Inhibitor

The HEK 293T cells were counted, seeded using SOL COUNT (SOL Inc.) into 1 × 10^7^ cells in 100-mm culture plate, and transfected with 2 μg of pCS4-Flag- hGH, pCS4-Flag- hGH (K67R), pCS4-Flag- hGH (K141R) and pCS4-Flag- hGH (K166R), respectively. At 24 h after the transfection, the cells were seeded into 60-mm culture plates, and treated with the protein synthesis inhibitor, cycloheximide (CHX) (Sigma-Aldrich) (100 μg/mL), and then the half-life of each protein was detected at 1, 2, 4 and 8 h after the treatment of the protein synthesis inhibitor. Furthermore, MG132 (Sigma-Aldrich) (5 μg/mL) was independently treated for 0, 1, 2 and 4 h. For Western blot analysis to detect hGH proteins, an anti-Flag (F3165, Sigma-Aldrich) antibody was used.

### 4.5. Purification of rhGH and AUT-rhGH

For untagged hGH, PCR was performed from pCS4-Flag-hGH, and pCS4-Flag-AUT-hGH using fwd 5’-GCGCCATGGCGATGTTCCCAACCATTCCCTTAT-3’ and rev 5’- GCGCTCGAGCTAGAAGCCACAGCTGCCCTC-3’, respectively. For cloning pET-28a (Novagen, Madison, WI, USA), Nco I and Xho I restriction enzyme sites were used. The nucleotide sequences of inserts were verified by direct sequencing (3730xl DNA Analyzers, Applied Biosystems, Waltham, MA, USA).

Subsequently, pET28a-hGH and pET28a-AUT-hGH were transformed into *E. coli* BL21 (DE3) cells for protein expression. A 25 mL aliquot of an overnight culture was seeded into 2 L of fresh LB medium containing 50 mg/mL kanamycin and grown at 37 °C to reach an OD_600_ absorbance of 0.6–0.8. Subsequently, protein expression was induced with 1 mM IPTG, and the cells were further cultured for another 16 h at 20 °C. After culture, the cells were harvested by centrifugation at 3,000 rpm, and cell pellets were suspended in lysis buffer (50 mM Tris-HCl (pH8.0), 10% glycerol) containing protease inhibitor cocktail (Roche, Barcelona, Spain) and sonication was applied at 4 °C. Subsequently, the lysate was centrifuged at 13,000 rpm, for 30 min at 4 °C, and soluble supernatants were obtained. For purification, soluble supernatants were loaded onto a 20 mL Hiprep DEAE FF 16/10 column (GE Healthcare, Chicago, IL, USA) and purified by anion exchange and gel filtration chromatography using AKTA Starter (GE Healthcare). All purification procedures were performed at 4 °C. Protein concentration was determined by the Bradford and bicinchoninic acid (BCA, Bio-Rad, Hercules, CA, USA) assays using bovine serum albumin (BSA) as a standard [44]. Protein purity was initially monitored by SDS-PAGE and silver staining.

### 4.6. Comparison of Structure of hGH and AUT-hGH

To compare the structure of hGH and AUT-hGH, an hGH pdb file was obtained from the RCSB protein data bank site (https://www.rcsb.org, accessed on 29 November 2018). The AUT-hGH pdb file was saved using a mutagenesis algorithm of the PyMOL program (The PyMOL Molecular Graphics System, Version 2.0 Schrödinger, LLC). The structure of AUT-hGH was compared with that of hGH using the Wincoot program (ver. 0.9.4.1) [32].

### 4.7. Signal Transduction by hGH and Its Mutant in Cells

To examine the signal transduction by hGH, AUT-hGH, commercial hGH (Saizen, Merck Serono, Darmstadt, Germany), NIH3T3 cells were counted, seeded using SOL COUNT (SOL Inc.) into 1 × 10^6^ cells in 60-mm culture plate, and starved in DMEM supplemented with 0.5% FBS for a day. The hGHs were then treated with 100 ng/mL for 2 days. After 2 days, Western blotting was performed to analyze the signal transduction in cells. Anti-STAT3 (sc-482, Santa Cruz), anti-phospho-STAT3 (9131S, Cell Signaling Technology, Danvers, MA, USA), anti-AKT (sc-8312, Santa Cruz), anti-phospho-AKT (9271S, Cell Signaling Technology), anti-ERK1/2 (LF-MA0134, Ab frontier), anti-phospho-ERK1/2 (LF-PA0090, Ab frontier), and anti-β-actin (sc-47778, Santa Cruz) were used for Western blot analysis.

### 4.8. Pharmacokinetic Study of AUT-hGH

#### 4.8.1. Animals and Treatment

The 8-week-old male SD rats were purchased from Orient Bio Co. Ltd. (Senongnam-si, Korea) and used after monitoring for a week. The experimental procedures were performed following the Biotoxtech (Cheongju, Korea) guidelines (experiment number B18854). Two to three rats were housed in every cage during monitoring and one rat per cage (260W × 350D × 210H (mm)) during the experiment. The animal groups consisted of negative control, hGH, AUT-hGH, and Saizen (as a comparative substance), and all substances were single subcutaneously administered, respectively. A single dose of hGH, AUT-hGH, and Saizen was set to 720 μg/kg. The normal and negative controls received injectable water, as an excipient. The volume of the solution was set at 1 mL/kg, and the volume of the solution was calculated based on the weight measured close to the day of administration.

#### 4.8.2. Blood Sampling

Blood samples were collected at 0, 1, 2, 4, 8, 12, 16, 24, 30 and 48 h after administration. About 0.5 mL of blood was collected from the jugular vein using a disposable syringe (1 mL, 25 G, Doowon Meditec Corp., Yongin, Korea) treated with heparin (Lot No.: 14030, Choongwae Pharma Corp., Seoul, Korea, 100 IU/mL) and placed in a microtube in a refrigerated state. To separate plasma, blood was separated into two microtubes (100 μL per sampling point) and rapidly centrifuged (12,000 rpm, 3 min, 4 °C, Micro 17TR, Hanil Science Industrial, Gimpo, Korea), and kept cooling in dry ice. The separated plasma was kept in an ultra-low temperature freezer (-80-60 °C, DFU-657CL, Operon, Gimpo, Korea) before analysis.

#### 4.8.3. ELISA for the Detection of hGH in Plasma

ELISA for hGH and AUT-hGH was performed using a Quantikine ELISA kit of hGH (RnD System, Minneapolis, MN, USA). The experiment was performed according to the provided instructions. The values represent the means ± SEM of the three independent experiments.

#### 4.8.4. Analysis for the Half-Life of AUT-hGH

Half-life analysis was performed as shown at the sites https://asan.shinyapps.io/pkrshiny/ (accessed on 28 May 2021) and https://www.aatbio.com/tools/ed50-calculator (accessed on 6 December 2018).

### 4.9. Ubiquitination of hGH in Blood

#### 4.9.1. Plasma Preparation and Pull-Down Assay Using Ni-NTA Beads

hGH-His was constructed by removing the stop codon from the aforementioned pET-28a-hGH using site-directed mutagenesis for PCR. The protein was purified by the same purification method as described above. In addition, then, to investigate whether hGH is ubiquitinated in the blood stream, 300 μL mouse whole blood was cleared for 1 h at 4 °C using 300 μL protein A/G Agarose (sc-2003, Santa Cruz), then centrifuged twice at 3000 rpm for 1 min to obtain supernatants. An amount of 100 ng of purified hGH-His was incubated for 1 h at 37 °C with MG132 (Sigma-Aldrich) (5 μg/mL) in 1.3 mL mouse whole blood and pull-down assay was performed using 30 μL Ni-NTA beads (Qiagen, Hilden, Germany). Then, the precipitant for hGH-His was washed five times with phosphate-buffered saline (PBS, Biosesang, Seongnam-si, Korea).

#### 4.9.2. SDS-PAGE Separation

Precipitant hGH-His was mixed with 5× SDS loading buffer and boiled for 10 min at 80 °C on a heat block. After centrifugation, each protein sample was applied on 12% SDS-PAGE. After visualization using Coomassie brilliant blue (Bio-Rad), the gel from each lane was cut into eight fractions and chopped into small pieces f or in-gel digestion.

#### 4.9.3. Enzymatic In-Gel Digestion

For the digestion, the gel pieces were washed with distilled water three times to remove SDS and dehydrated using 100% acetonitrile (ACN). Protein reduction was performed using 10 mM dithiothreitol in 50 mM NH_4_HCO_3_ for 45 min at 56 °C. After washing with 100% ACN, alkylation of cysteines was done with 55 mM iodoacetamide in 50 mM NH_4_HCO_3_ for 30 min in the dark. Finally, each dehydrated gel piece was treated with 12.5 ng/µL sequencing grade modified trypsin (Promega, Madison, WI, USA) in 50 mM NH_4_HCO_3_ buffer (pH 7.8) at 37 °C for overnight. Following digestion, tryptic peptides were extracted with 5% formic acid in 50% ACN solution at room temperature for 20 min. The supernatants were collected and dried with SpeedVac. Before MS analysis, the samples suspended in 0.1% formic acid were purified and concentrated using C18 ZipTips (Millipore, Burlington, MA, USA).

#### 4.9.4. Nano-LC-ESI-MS/MS Analysis

Peptide separation was performed using Dionex UltiMate 3000 RSLCnano system (Thermo-Fisher Scientific, Waltham, MA, USA). Tryptic peptides from bead column were reconstituted using 0.1% formic acid and separated on a 50-cm Easy-Spray column with a 75-μm inner diameter packed with 2 μm C18 resin (Thermo-Fisher Scientific) over 120 min (300 nL/min) using a 0 to 45% ACN gradient in 0.1% formic acid at 50 °C. The LC was coupled to a Q Exactive Plus BioPharm mass spectrometer with a nano-ESI source (Thermo-Fisher Scientific). Mass spectra were acquired in a data-dependent mode with an automatic switch between a full scan and five data-dependent MS/MS scans. The target value for the full scan MS spectra was 3,000,000 with a maximum injection time of 120 ms and a resolution of 70,000 at m/z 400. The ion target value for MS/MS was set to 1,000,000 with a maximum injection time of 120 ms and a resolution of 17500 at m/z 400. Dynamic exclusion of repeated peptides was applied for 20 s.

#### 4.9.5. Database Searching

The resulting raw files were processed using Proteome Discoverer (version 2.3, Thermo-Fisher Scientific) for identification with the database of Homo sapiens (organism ID: 9606, 71567 entries, UniProt). The search parameters were set as default including cysteine carbamidomethylation as a fixed modification, N-terminal acetylation, methionine oxidation phospho-serine, -threonine and -tyrosine as variable modifications and di-glycine modification at lysine residue with two miscleavages. Peptide identification was based on a search with an initial mass deviation of the precursor ion of up to 10 ppm, and the allowed fragment mass deviation was set to 20 ppm.

#### 4.9.6. Polyubiquitination of hGH in the Blood Stream

hGH-His was incubated with and without MG132 (Sigma-Aldrich) (5 μg/mL). As previously explained, pull-down assay was performed. In addition, then, Western blotting was performed using an anti-ubiquitin antibody (sc-8017, Santa Cruz) to examine the polyubiquitinated hGH in the blood stream.

### 4.10. Efficacy Study of AUT-hGH

#### 4.10.1. Animals and Treatment

Rats with their pituitary gland removed at 4 weeks of age and normal rats were purchased from Orient Bio Inc. and used after monitoring for a week. The experimental procedures were performed following the guideline of Biotoxtech Inc. (experiment number B17622). The animal groups consisted of the negative and positive controls, hGH, and AUT-hGH. All substances were administered on a daily basis, and in intervals of 3 days and 7 days, repeatedly. The single dose of hGH and AUT-hGH was set to 0.44 IU/kg.

#### 4.10.2. Measurement of Length for Growth Plate

Before tibial extraction, tetracycline hydrochloride (Lot No.: SLBQ2368V, Sigma-Aldrich) was administered intraperitoneally at 20 mg/kg for 72 h. It was prepared and used in a physiological saline injection solution (Lot No.: GAA6023, JW Pharmaceutical Co., Ltd., Korea) at 4 mg/mL. Then the collected tibia was fixed in 4% paraformaldehyde (Lot No.: #STBG9973, Sigma-Aldrich) for 48 h and then 10% ethylene diamine tetra acetic acid (Lot No.: E0008RG1, Daejung Chemicals & Metals Co., Ltd., Korea) solution and demineralized for 24 h. The tibia after demineralization was dehydrated for 24 h by putting it in 30% sucrose (Lot No.: #SLBR5401V, Sigma-Aldrich) solution. Next, the tibia was cut to a thickness of 40 μm in the longitudinal direction (longitudinally) using a cryosection machine (CM3050S, Leica, Germany) and attached to the slide. The prepared frozen section slide was sealed with Fluormount, observed with a fluorescence microscope (Observer Z1, Carl Zeiss, Germany) and photographed with a tissue (Axiovision V.4.6, Carl Zeiss) to measure the length between the bands produced by tetracycline in the growth plate.

## Supplementary Materials

Supplementary materials can be found at https://www.mdpi.com/article/10.3390/ijms22126268/s1.

## Abbreviations

AUT	Anti-ubiquitination technology
CHX	Cycloheximide
DEAE	Diethylaminoethyl cellulose
DMEM	Dulbecco’s modified Eagle’s medium
E1	Ubiquitin-activating
E2 E3	Ubiquitin-conjugating Ubiquitin-ligase
ELISA	Enzyme-linked immunosorbent assay
FBS	Fetal bovine serum
hGH IPTG	Human growth hormone Isopropyl-b-D-thiogalactopyranoside
Nano-LC-ESI-MS/MS	Nano liquid chromatography-tandem mass spectrometry
PMSF SDS-PAGE	Phenylmethanesulfonyl fluoride Sodium dodecyl sulfate-polyacrylamide gel electrophoresis
