# Supplementary material for: Long-Lasting Growth Hormone Regulated by the Ubiquitin-Proteasome System
Source: Int J Mol Sci. 2021 Jun 10;22(12):6268. doi: 10.3390/ijms22126268 (PMC8230561; doi:10.3390/ijms22126268)
Supplement: Supplementary file 1 [file ijms-22-06268-s001.zip › ijms-1194619-supplementary.pdf]

Supple Figure S1

A

ATG GCT ACA GGC TCC CGG ACG TCC CTG CTC CTG GCT TTT GGC CTG CTC TGC CTG CCC TGG CTT CAA GAG
GGC AGT GCC TTC CCA ACC ATT CCC TTA TCC AGG CTT TTT GAC AAC GCT ATG CTC CGC GCC CAT CGT CTG
CAC CAG CTG GCC TTT GAC ACC TAC CAG GAG TTT GAA GAA GCC TAT ATC CCA AAG GAA CAG **AAG** TAT TCA
TTC CTG CAG AAC CCC CAG ACC TCC CTC TGT TTC TCA GAG TCT ATT CCG ACA CCC TCC AAC AGG GAG GAA
ACA CAA CAG AAA TCC AAC CTA GAG CTG CTC CGC ATC TCC CTG CTG CTC ATC CAG TCG TGG CTG GAG CCC
GTG CAG TTC CTC AGG AGT GTC TTC GCC AAC AGC CTG GTG TAC GGC GCC TCT GAC AGC AAC GTC TAT GAC
CTC CTA **AAG** GAC CTA GAG GAA GGC ATC CAA ACG CTG ATG GGG AGG CTG GAA GAT GGC AGC CCC CGG ACT
GGG CAG ATC TTC **AAG** CAG ACC TAC AGC AAG TTC GAC ACA AAC TCA CAC AAC GAT GAC GCA CTA CTC AAG
AAC TAC GGG CTG CTC TAC TGC TTC AGG AAG GAC ATG GAC AAG GTC GAG ACA TTC CTG CGC ATC GTG CAG
TGC CGC TCT GTG GAG GGC **AGC TGT GGC TTC TAG**

B **hGH**

ATG TTC CCA ACC ATT CCC TTA TCC AGG CTT TTT GAC AAC GCT ATG CTC CGC GCC CAT CGT CTG CAC CAG
CTG GCC TTT GAC ACC TAC CAG GAG TTT GAA GAA GCC TAT ATC CCA AAG GAA CAG AAG TAT TCA TTC CTG
CAG AAC CCC CAG ACC TCC CTC TGT TTC TCA GAG TCT ATT CCG ACA CCC TCC AAC AGG GAG GAA ACA CAA
CAG AAA TCC AAC CTA GAG CTG CTC CGC ATC TCC CTG CTG CTC ATC CAG TCG TGG CTG GAG CCC GTG CAG
TTC CTC AGG AGT GTC TTC GCC AAC AGC CTG GTG TAC GGC GCC TCT GAC AGC AAC GTC TAT GAC CTC CTA
AAG GAC CTA GAG GAA GGC ATC CAA ACG CTG ATG GGG AGG CTG GAA GAT GGC AGC CCC CGG ACT GGG CAG
ATC TTC AAG CAG ACC TAC AGC AAG TTC GAC ACA AAC TCA CAC AAC GAT GAC GCA CTA CTC AAG AAC TAC
GGG CTG CTC TAC TGC TTC AGG AAG GAC ATG GAC AAG GTC GAG ACA TTC CTG CGC ATC GTG CAG TGC CGC
TCT GTG GAG GGC AGC TGT GGC TTC **TAG**

AUT-hGH

ATG TTC CCA ACC ATT CCC TTA TCC AGG CTT TTT GAC AAC GCT ATG CTC CGC GCC CAT CGT CTG CAC CAG
CTG GCC TTT GAC ACC TAC CAG GAG TTT GAA GAA GCC TAT ATC CCA AAG GAA CAG AAG TAT TCA TTC CTG
CAG AAC CCC CAG ACC TCC CTC TGT TTC TCA GAG TCT ATT CCG ACA CCC TCC AAC AGG GAG GAA ACA CAA
CAG AAA TCC AAC CTA GAG CTG CTC CGC ATC TCC CTG CTG CTC ATC CAG TCG TGG CTG GAG CCC GTG CAG
TTC CTC AGG AGT GTC TTC GCC AAC AGC CTG GTG TAC GGC GCC TCT GAC AGC AAC GTC TAT GAC CTC CTA
AGG GAC CTA GAG GAA GGC ATC CAA ACG CTG ATG GGG AGG CTG GAA GAT GGC AGC CCC CGG ACT GGG CAG
ATC TTC AAG CAG ACC TAC AGC AAG TTC GAC ACA AAC TCA CAC AAC GAT GAC GCA CTA CTC AAG AAC TAC
GGG CTG CTC TAC TGC TTC AGG AAG GAC ATG GAC AAG GTC GAG ACA TTC CTG CGC ATC GTG CAG TGC CGC
TCT GTG GAG GGC AGC TGT GGC TTC **TAG**

Supple Table S1

Ubiquitination at K141 of hGH in serum

#1	b^+	b^{2+}	Seq.	y^+	y^{2+}	#2
1	88.03930	44.52329	S			21
2	187.10772	94.05750	V	2289.13975	1145.07351	20
3	334.17613	167.59170	F	2190.07134	1095.53931	19
4	405.21325	203.11026	A	2043.00292	1022.00510	18
5	519.25617	260.13173	N	1971.96581	986.48654	17
6	606.28820	303.64774	S	1857.92288	929.46508	16
7	719.37227	360.18977	L	1770.89086	885.94907	15
8	818.44068	409.72398	V	1657.80679	829.40703	14
9	981.50401	491.25564	Y	1558.73838	779.87283	13
10	1038.52547	519.76637	G	1395.67505	698.34116	12
11	1109.56259	555.28493	A	1338.65359	669.83043	11
12	1196.59461	598.80095	S	1267.61647	634.31187	10
13	1311.62156	656.31442	D	1180.58444	590.79586	9
14	1398.65359	699.83043	S	1065.55750	533.28239	8
15	1512.69651	756.85189	N	978.52547	489.76637	7
16	1611.76493	806.38610	V	864.48254	432.74491	6
17	1774.82826	887.91777	Y	765.41413	383.21070	5
18	1889.85520	945.43124	D	602.35080	301.67904	4
19	2002.93926	1001.97327	L	487.32386	244.16557	3
20	2116.02333	1058.51530	L	374.23980	187.62354	2
21			K-GG	261.15573	131.08150	1

Supple Table S2

Lysine(K) residue position	The substitutes of hGH with lysine converted to arginine
67	pCS4-Flag-hGH (K67R)
141	pCS4-Flag-hGH (K141R)
166	pCS4-Flag-hGH (K166R)
